# High divergence of human astrovirus genotypes circulating in pediatric patients hospitalized with acute gastroenteritis in Chiang Mai, Thailand, 2017–2020

**DOI:** 10.1038/s41598-021-02745-7

**Published:** 2021-12-01

**Authors:** Hongyu Wei, Pattara Khamrin, Kattareeya Kumthip, Arpaporn Yodmeeklin, Niwat Maneekarn

**Affiliations:** 1 Department of Microbiology, Faculty of Medicine, Chiang Mai University, Chiang Mai, 50200 Thailand; 2 Center of Excellence in Emerging and Re-Emerging Diarrheal Viruses, Chiang Mai University, Chiang Mai, 50200 Thailand; 3 Youjiang Medical University for Nationalities, Baise, 533000 Guangxi China

**Keywords:** Viral epidemiology, Gastrointestinal models, Sequencing

## Abstract

Human astrovirus (HAstV) is one of the common causes of acute gastroenteritis in children. The investigation of molecular epidemiology of HAstV is essential for monitoring the emergence and/or re-emergence of new HAstV genotypes, as well as understanding the evolution of HAstV circulating in children suffering from acute gastroenteritis. The present study aimed to investigate the prevalence and distribution of HAstVs strains circulating in children hospitalized with acute gastroenteritis in Chiang Mai, Thailand during 2017–2020. A total of 1500 fecal specimens collected from children with acute gastroenteritis were screened for HAstV by RT-PCR that targeted the partial RdRp in ORF1b and strains were characterized by sequencing and phylogenetic analysis. Of the 1500 fecal samples, 39 (2.6%) were positive for HAstV. Of these, both classic and novel HAstV genotypes, including classic HAstV1–HAstV5, novel HAstV-MLB1, MLB2, and HAstV-VA2, were detected. The data in this study revealed a high divergence of HAstV genotypes circulating in pediatric patients admitted to the hospitals with acute gastroenteritis in Chiang Mai, Thailand during 2017–2020.

## Introduction

Human astrovirus (HAstV) infection is one of the leading causes of acute gastroenteritis, mostly in young children, elderly people, and immunocompromised patients^1,2^. The HAstV infection results in diseases ranging from asymptomatic^3^ to mild watery diarrhea^4^ and systemic diseases^5^. Currently, the genotype of HAstV has been classified into classic HAstV (HAstV1–HAstV8), novel HAstV-MLB (MLB1–MLB3), and novel HAstV-VA/HMO (VA1–VA5)^6^. The classic HAstV accounts for 2.9–5.0% of acute gastroenteritis in children, and classic HAstV1 remains the predominant genotype^7^. Although the novel HAstV-MLB and HAstV-VA/HMO were initially detected in children with gastroenteritis, the definite association between these novel HAstV genotypes and gastroenteritis has not yet been established^8,9^. On the other hand, the novel HAstV-MLB and HAstV-VA have been increasingly reported to associate with central nervous system infection in humans, particularly, in immunocompromised individuals^5,7,10,11^.

The molecular epidemiology study of HAstV in Chiang Mai, Thailand during 2000–2016 reported the detections of classic HAstV1, HAstV4, HAstV5, HAstV8, novel HAstV-MLB1, MLB2, and novel HAstV-VA2 in pediatric patients with acute gastroenteritis^12,13^. The aim of this study was to conduct a follow-up surveillance to monitoring the HAstV genotypes circulating in children admitted to the hospitals with acute gastroenteritis in Chiang Mai, Thailand during 2017–2020.

## Results

### Prevalence of HAstV infection

Of 1500 stool samples tested, 39 were positive for HAstV. The overall prevalence of HAstV infection spanning from 2017 to 2020 was 2.6% (39 of 1500). When the prevalence of HAstV infection was analysed on a yearly basis, it was found that the prevalence was 3.1% (8 of 257) in 2017, 3.2% (20 of 620) in 2018, 1.5% (7 of 473) in 2019, and 2.7% (4 of 150) in 2020 as shown in Table 1. Among 39 stool samples that were positive for HAstV, approximately half of them were co-infected with many other enteric viruses at a rate of 51.3% (20 of 39). Of these, 18 were co-infected either with rotavirus, norovirus, adenovirus, sapovirus, or enterovirus, while the other two samples were co-infected with two or more enteric viruses, namely one sample was co-infected with rotavirus and norovirus, and another sample was co-infected with adenovirus, norovirus, and enterovirus (Table 1).

**Table 1:** Prevalence of HAstV infection and co-infection in children hospitalized with acute gastroenteritis in Chiang Mai, Thailand during 2017–2020.

Years	No. of specimen tested	No. of HAstV positive (%)	No. of HAstV single infection (%)	No. of HAstV co-infection (%)	No. and patterns of HAstV co-infection				
					RV	NoV	SaV	EV	≥ 2 viruses^a^
2017	257	8 (3.1)	3 (37.5)	5 (62.5)	–	1	1	1	2
2018	620	20 (3.2)	9 (45.0)	11 (55.0)	8	1	–	2	–
2019	473	7 (1.5)	5 (71.4)	2 (28.6)	–	2	–	–	–
2020	150	4 (2.7)	2 (50.0)	2 (50.0)	1	1	–	–	–
Total	1500	39 (2.6)	19 (48.7)	20 (51.3)	9	5	1	3	2

*RV* rotavirus, *NoV* norovirus, *AdV* adenovirus, *EV* enterovirus, *SaV* sapovirus.

^a^HAstV co-infection with other two or more enteric viruses; RV + NoV (n = 1); AdV + NoV + EV (n = 1).

### Prevalence of HAstV infection in patients with different age groups

**Figure 1:**
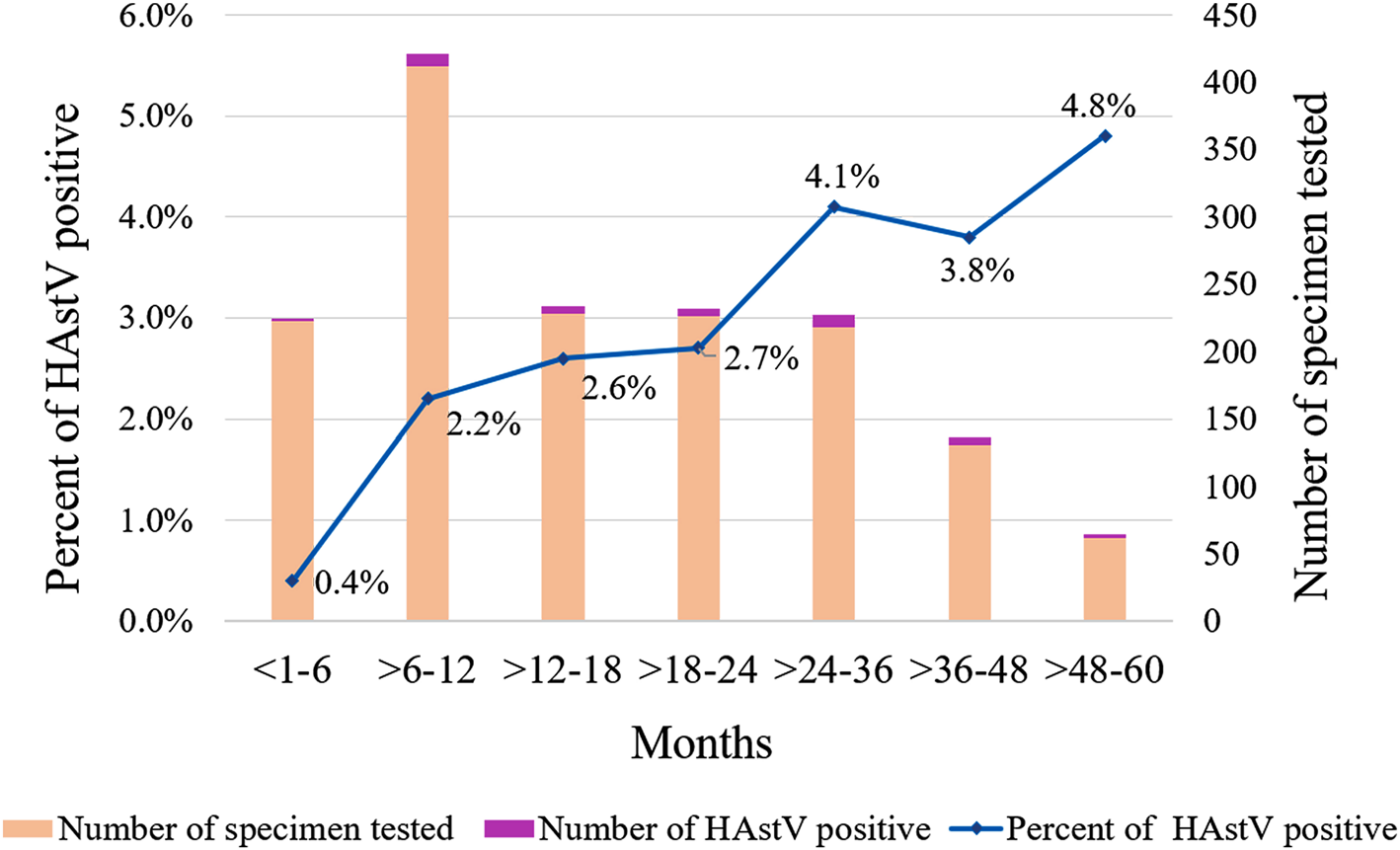
Prevalence of HAstV infection in children with acute gastroenteritis of different age groups.

In order to analyse the prevalence of HAstV infection on the basis of demographic characteristics, the patients were stratified into seven different age groups, < 1–6, > 6–12, > 12–18, > 18–24, > 24–36, > 36–48, and > 48–60 months old. It was found that the prevalence of HAstV was highest at 4.8% (3 of 62) at the age group of > 48–60 months old, followed by 4.1% (9 of 218), 3.8% (5 of 131), 2.7% (6 of 226), 2.6% (6 of 228), 2.2% (9 of 412), and 0.4% (1 of 223) at the age groups of > 24–36, > 36–48, > 18–24, > 12–18, > 6–12, and < 1–6 months old, respectively, as shown in Fig. 1. However, the difference of HAstV infection rates in different age groups was not statistically significant (p value > 0.05) except for those of the group of < 1–6 months old that was significantly different from those of > 18–24, > 24–36, > 36–48, and > 48–60 months old with the p values of 0.049, 0.009, 0.016, and 0.008, respectively.

### Distribution of HAstV genotypes

In this study, all three major clades of HAstV, including classic HAstV, novel HAstV-MLB, and novel HAstV-VA were detected in children hospitalized with acute gastroenteritis. The classic HAstV1 was the most common genotype with the prevalence of 1.46% (22 of 1500), followed by novel HAstV-MLB1 0.4% (6 of 1500), classic HAstV4 at 0.2% (3 of 1500), classic HAstV3, HAstV5, and novel HAstV-MLB2 each at the prevalence of 0.13% (2 of 1500), whereas the classic HAstV2 and novel HAstV-VA2 each of which was detected at the prevalence of 0.06% (1 of 1500) (Table 2).

**Table 2:** Distribution of HAstV genotypes in children hospitalized with acute gastroenteritis in Chiang Mai, Thailand during 2017–2020.

Years	No. of specimen tested	No. of human astrovirus genotype positive (%)								Total
		HAstV1	HAstV2	HAstV3	HAstV4	HAstV5	MLB1	MLB2	VA2	
2017	257	2	–	–	1	1	2	1	1	8
2018	620	16	–	1	–	–	2	1	–	20
2019	473	2	–	–	2	1	2	–	–	7
2020	150	2	1	1	–	–	–	–	–	4
Total	1500	22 (1.46)	1 (0.06)	2 (0.13)	3 (0.2)	2 (0.13)	6 (0.4)	2 (0.13)	1 (0.06)	39 (2.6)

### Distribution of HAstV genotype infection in children with different age groups

Analysis of the patterns of HAstV genotype infections in children with different age groups revealed that a greater variety of HAstV genotypes with high infection rates circulated in older children were higher compared with younger age groups as shown in Table 3. However, significant differences in an overall prevalence of HAstV infections were observed only when the prevalence of a group of < 1–6 months old was compared with those of > 18–24, > 24–36, > 36–48, and > 48–60 months old with the p values of 0.049, 0.009, 0.016, and 0.008, respectively, whereas when compared with those of > 6–12 and > 12–18 months old a difference was not significant (p value 0.082 and 0.056, respectively). The overall prevalence of pairwise comparisons between all other age groups were not statistically significant (p value ranged from 0.174 to 0.947). It was interesting to note that HAstV1 was found to infect the children in all age groups with a prevalence of 1.5%. Pairwise comparisons of HAstV1 prevalence in different age groups were not statistically significant (p values ranged from 0.093 to 0.927). Furthermore, pairwise comparisons in different age groups of the prevalence of other HAstV genotypes detected in this study, including HAstV2, HAstV3, HAstV4, HAstV5, MLB1, MLB2, and VA were also not statistically significant.

**Table 3:** Distribution of HAstV genotypes in different age groups of children with acute gastroenteritis in Chiang Mai, Thailand during 2017–2020.

Age groups (months)	No. of specimen tested	No. of HAstV positive (%)	HAstV genotypes (%)							
			HAstV1	HAstV2	HAstV3	HAstV4	HAstV5	MLB1	MLB2	VA2
< 1–6	223	1 (0.4)*	1 (0.4)	–	–	–	–	–	–	–
> 6–12	412	9 (2.2)	8 (1.9)	–	–	–	–	–	1 (0.2)	–
> 12–18	228	6 (2.6)	5 (2.2)	–	–	–	–	1 (0.4)	–	–
> 18–24	226	6 (2.7)*	3 (1.3)	–	1 (0.4)	–	–	2 (0.9)	–	–
> 24–36	218	9 (4.1)*	3 (1.4)	1 (0.5)	–	2 (0.9)	1 (0.5)	2 (0.9)	–	–
> 36–48	131	5 (3.8)*	1 (0.8)	–	1 (0.8)	1 (0.8)	1 (0.8)	–	–	1 (0.8)
> 48–60	62	3 (4.8)*	1 (1.6)	–	–	–	–	1 (1.6)	1 (1.6)	–
Total	1500	39 (2.6)	22 (1.5)	1 (0.06)	2 (0.1)	3 (0.2)	2 (0.1)	6 (0.4)	2 (0.1)	1 (0.06)

p value of < 1–6 vs > 18–24 = 0.049*; < 1–6 vs > 24–36 = 0.009*; < 1–6 vs > 36–48 = 0.016*; < 1–6 vs > 48–60 = 0.008*

*p value equal to or less than 0.05 (≤ 0.05) is statistically significant.

### Phylogenetic analysis

**Figure 2:**
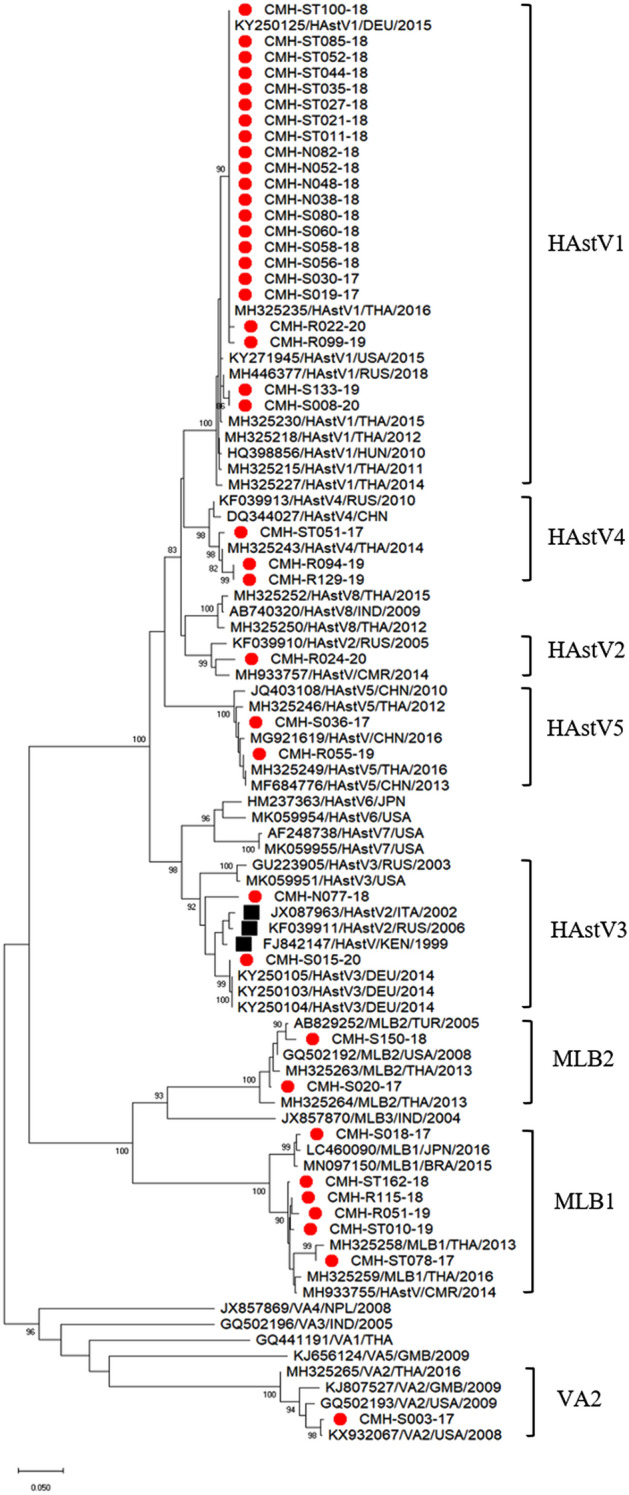
Phylogenetic tree of HAstV strains detected in Chiang Mai, Thailand during the period of 2017–2020. The scale bar represents nucleotide substitution per site. The HAstV strains detected in this study are labeled by red dot, HAstV recombinant reference strains (ORF1b: genotype 3/ORF2: genotype 2) are labeled by black square. The HAstV reference strains obtained from the GenBank database are indicated by accession number.

The partial RdRp nucleotide sequence (342 bp) of 39 HAstV strains were phylogenetically analysed together with the reference strains as shown in Fig. 2. Twenty-two strains of classic HAstV1 detected in this study, even though they were detected in the specimens collected in different years, were relatively homogenous. These HAstV1 strains showed the nucleotide sequence identities among themselves ranging from 97.5 to 100% and were closely related to the classic HAstV1 reference strains reported previously from Hungary (HQ398856), Thailand (MH325235, MH325215, MH325230, MH325218, and MH325227), USA (KY271945), Russia (MH446377), and Germany (KY250125) with the nucleotide sequence identities ranging from 97.2 to 100%. The only strain of classic HAstV2 detected in this study was closely related to the classic HAstV2 reference strains previously reported from Russia (KF039910) and Cameroon (MH933757) with the nucleotide sequence identities of 95.7% and 96.3%, respectively. Two strains of classic HAstV3, one (CMH-S15-20) was most closely related to the classic HAstV3 reference strains reported from Germany (KY25013, KY250104, and KY250105) with the nucleotide sequence identities ranging from 99.3 to 99.6%, whereas another one (CMH-N77-18) was most closely related to the reference strains of classic HAstV2 genotype (indicated by black square) which were reported previously from Italy (JX087963), Russia (KF039911), and Kenya (FJ842147) with the nucleotide sequence identities ranging from 93.8 to 96.6%. However, in the phylogenetic tree, these HAstV2 reference strains and CMH-N77-18 strain clustered together with other classic HAstV3 reference strains. Of note, the genotypes of these strains (JX087963, KF039911, and FJ842147) were assigned based on their complete genome sequences which belonged to classic HAstV2 genotype. In fact, these strains are the recombinant strains with RdRp (ORF1b) of HAstV3 genotype and ORF2 of HAstV2 genotype^14–16^. This is the reason to explain why these classic HAstV2 reference strains clustered within the HAstV3 genotypes in the phylogenetic tree of RdRp sequences. Three strains of classic HAstV4 were most closely related to the classic HAstV4 reference strains reported previously from Thailand (MH325243), Russia (KF039913), and China (DQ344027) with the nucleotide sequence identities ranging from 96.3 to 99.0%. Two strains of classic HAstV5 were most closely related to the classic HAstV5 reference strains reported from Thailand (MH325246 and MH325249) and China (JQ403108, MG921619, and MF684776) with the nucleotide sequence identities ranging from 96.9 to 99.0%.

Six strains of novel HAstV-MLB1 detected in the present study were most closely related to the novel HAstV-MLB1 reference strains reported from Thailand (MH325258 and MH325259), Japan (LC460090), Brazil (MN097150), and Cameroon (MH933755) with the nucleotide sequence identities ranging from 93.4 to 99.0%. Two strains of novel HAstV-MLB2 were most closely related to the novel HAstV-MLB2 reference strains reported previously from Thailand (MH325263 and MH325264) with the nucleotide sequence identities ranging from 96.2 to 99.0%. The only one strain of novel HAstV-VA2 detected in this study was most closely related to the novel HAstV-VA2 reference strains reported from the USA (GQ502193 and KX932067) with the nucleotide sequence identities ranging from 96.8 to 99.4%.

## Discussion

The prevalence of HAstV infection has been sequentially reported from around the world with a wide range of variability from 0 to 29.7%^7^. The prevalence varies from study to study depending on the study population and geographical region that the study has been conducted. For instance, in South America, the prevalence of HAstV infection in Brazil was reported with low infection rate at 0.8% ^17^, whereas in North America a prevalence was reported at 3.5% in Mexico^18^. In Africa, a prevalence of HAstV infection was reported at 7.0% in South Africa^19^, 9.9% in Kenya and Gambia^3^, 10.3% in Congo^20^, 11–14% in Egypt^21,22^, and 19.4% in Nigeria^23^. In Europe, a prevalence of HAstV infection was reported at 2.5% in Netherland^24^ and 3% in Italy^25^. In Asia, a prevalence of HAstV infection was reported at 1.6–2.8% in China^26,27^, 1.9% in Korea^28^, 2.6% in Taiwan^29^, 2.4–16.4% in Japan^30,31^, and 1.4–3.1% in Thailand^12,13,32,33^. In the present study, a prevalence of HAstV infection in children with acute gastroenteritis in Chiang Mai, Thailand during the period of 2017–2020 was 2.6% which was exactly the same as those reported previously from the same geographical area during 2011–2016^13^. The data imply that in a past decade (2011–2020) HAstV was circulating in children with acute gastroenteritis in Thailand with a relatively constant infection rate of approximately 2.6%. However, when we analyzed a prevalence on a yearly basis, it varies year by year from 1.5–5.0% during 2011–2016^13^ and 1.5–3.2% during 2017–2020 in this study. In addition, it is interesting to note that HAstV infections observed in this study were co-infected with many other enteric viruses, including rotavirus, norovirus, adenovirus, enterovirus, and sapovirus to as high as 51.3% (Table 1). Some of HAstV-infected cases were found to be co-infected with two or three other enteric viruses. This observation is similar to what we have reported previously during 2011–2016 at 57.4% in the same geographical area^13^, suggesting that gastroenteritis in children in Thailand are infected with a wide variety of enteric viruses and require more attention on the situation. Nevertheless, high rate of co-infection with other enteric viruses is not uncommon, it has also been reported elsewhere, such as 38.2% in Nigeria^23^ and 71% in Germany^34^. As expected and shown in Table 1 that the rotavirus was found to be the most common virus that co-infected with HAstV infection since rotavirus is the most common virus that causes gastroenteritis in children worldwide^35,36^.

Analysis of a prevalence of HAstV infection in children with different age groups (Fig. 1) revealed that the lowest infection rate at 0.4% was observed in children with the age of < 1–6 months old. Then, the infection rate abruptly increased to 2.2% in children with the age of > 6–12 months old and continued increasing in the older age groups, and reached highest rate in children with the age of > 48–60 months old. The data imply that at the age of < 1–6 months old, the children might be protected by maternal antibodies acquired in utero or via breast feeding and these maternal antibodies declines after > 6–12 months old. As a result, the infection rates went up to 2.2%, 2.6%, and 2.7% in children with the age of > 6–12, > 12–18, and > 18–24 months old, respectively. In addition, the infection rates increased further approximately two folds to 4.1%, 3.8%, and 4.8% in children with the ages of > 24–36, > 36–48, and > 48–60 months old. The infection rates increased approximately two folds in children with the age of > 24–60 months old compared to those observed in children with the age of > 6–24 months old is probably related to the hygienic fed pattern in younger children compared to relatively unhygienic behavior or activities at the playground of older children. Apparently, older children may have much more chances to expose to and being infected with HAstV as well as many other enteric viruses.

In this study, all three major clades of HAstV, including classic HAstV, novel HAstV-MLB, and novel HAstV-VA were detected in children hospitalized with acute gastroenteritis (Table 2). Our data demonstrate the divergence of classic HAstV genotypes, HAstV1-HAstV5, and novel HAstV-MLB1, MLB2, and VA2, and also reinforcing the most predominance of HAstV1 that has been reported from several countries around the world^7,37^, including Thailand^12,13,33^. In Chiang Mai, Thailand, it is noteworthy to mention that classic HAstV2 and HAstV3 were detected in 2001 and 2000, respectively, and both of them disappeared during 2002–2016^12,13^. In the present study, HAstV2 continued to disappear from 2017 to 2019 and was detected in 2020 whereas HAstV3 was detected in 2018 and 2020 but not in 2017 and 2019 (Table 2). The data indicate that the HAstV genotypes circulating in children with acute gastroenteritis in Chiang Mai, Thailand have changed over time and the data also demonstrate a great diversity of classic HAstV genotypes circulating in children with acute gastroenteritis in the past 4 years (2017–2020). Nevertheless, novel HAstV-MLB1, MLB2, and VA2 which were detected in our previous study^13^ remain detectable in this study and novel HAstV-MLB1 was detected as the second most common genotype. Analysis of the distribution of HAstV genotypes in children with different age groups revealed that classic HAstV1 infection was detected in all age groups analyzed in this study (Table 3), it is worthwhile to point out that children with the age of < 1–6 months were infected only with classic HAstV1 whereas children with older age were infected with diverse genotypes of two to three and five genotypes at the age of > 6–24 and > 24–48 months old, respectively. The data, again, confirm and reinforce the association of different rate of HAstV infection in different age groups in relation to acquired maternal antibody protection in children with the age of < 1–6 months old, hygienic fed pattern in children with the age of > 6–24 months old, and unhygienic behavior or activities at the playground that led to higher chance of exposure to and being infected with a wide range of HAstV genotypes in older children with the age of > 24–48 months old. Of note, the children with the age of > 48–60 months old were infected only with three genotypes of HAstV might probably be due to smaller sample size (62 cases) in this group compared to 218 and 131 cases in the age groups of > 24–36 and > 36–48 months old, respectively.

Phylogenetic analyses of the RdRp (ORF1b) nucleotide sequences of HAstV strains detected in this study revealed that these strains share high nucleotide sequence identities with global strains reported from different remote countries and continents, demonstrating a worldwide circulation of HAstV strains that occurs continuously and has an impact on epidemiology and evolution of HAstV strains.

In conclusion, this study provides additional epidemiology data and molecular characteristics of HAstV genotypes circulating in pediatric patients admitted to the hospitals with acute gastroenteritis in Chiang Mai, Thailand during the period of 2017–2020. Wide variety of classic and novel HAstV genotypes have been detected and analyzed phylogenetically. The data have an impact on a better understanding of molecular epidemiology and evolution of HAstV strains in Thailand.

## Materials and methods

### Ethics statement

The study protocols were carried out in accordance with relevant guidelines and regulations, and were approved by the Institutional Ethics Committee of the Faculty of Medicine, Chiang Mai University, Thailand (Approval number: MIC-2557-02710). Written informed consents were obtained from the participants’ guardian.

### Screening for diarrheal viruses

In addition to investigation for HAstV, the stool specimens included in this study were also screened for several other diarrheal viruses, including rotavirus (RV), norovirus (NoV), sapovirus (SaV), and enterovirus (EV) by using RT-PCR methods and adenovirus (AdV) by PCR as described previously^38–41^ in order to investigate a situation of coinfections of HAstV by several other diarrheal viruses.

### Specimen collection

A total of 1500 stool specimens were collected from pediatric patients under 5 years old admitted to the hospitals with acute gastroenteritis in Chiang Mai from 2017 to 2020. All samples were stored at − 20 °C until use.

### Detection of human astrovirus

The HAstV genome was extracted from 10% stool suspension in phosphate buffered saline (PBS) by using Geneaid viral genomic extraction kit (Geneaid Biotech, Taiwan) following the manufacturer’s instruction. The viral cDNA was synthesized using RevertAid First Strand cDNA Synthesis Kit (Thermo Scientific, USA). The astrovirus was detected by amplifying the RNA dependent RNA polymerase (RdRp) region of viral genome using forward primer SF0073 (5′-GATTGGACTCGATTTGATGG-3′) and reverse primer SF0076 (5′-CTGGCTTAACCCACATTCC-3′)^42^ by using the DreamTaq Green PCR Master Mix (2X) (Thermo Scientific, USA). The expected PCR product size of 409 bp was detected by electrophoresis on 1.5% agarose gel, stained with nucleic acid staining solution (RedSafe, iNtRON Biotechnology, South Korea), and visualized under ultraviolet light.

### Nucleotide sequencing and phylogenetic analysis

The HAstV strains detected in this study were further characterized for their genotypes by nucleotide sequencing. The PCR products were purified by using GenepHlow™ Gel/PCR kit (Geneaid Biotech, Taiwan). Then, the purified PCR products were sequenced (First Base Laboratory SDNBHN Selangor Darul Ehsan, Malaysia). The obtained nucleotide sequences were analysed by comparing with those of the reference strains available in the GenBank database using the Basic Local Alignment Search Tool (BLAST) server (https://blast.ncbi.nlm.nih.gov/Blast.cgi). The phylogenetic tree of the partial RdRp gene was constructed by using the MEGA X software based on the maximum likelihood method and selected the best-fit evolutionary model for the data set via Tamura-3-parameter model with 1000 replicates^43,44^.

### Statistical analysis

The MedCalc and IBM SPSS v. 22.0 software were used for statistical analysis.

The proportions and differences in the prevalence and genotype distribution in children with different age groups were statistically analyzed by using the MedCalc and Fisher’s exact test, respectively. The p value equal to or less than 0.05 (≤ 0.05) was statistically significant.

### Nucleotide sequence accession number

The nucleotide sequences of astroviruses detected in this study have been deposited in the GenBank database under the accession numbers MZ327095-MZ327133.
