# Author Correction: High divergence of human astrovirus genotypes circulating in pediatric patients hospitalized with acute gastroenteritis in Chiang Mai, Thailand, 2017–2020

**DOI:** 10.1038/s41598-021-03256-1

**Published:** 2021-12-07

**Authors:** Hongyu Wei, Pattara Khamrin, Kattareeya Kumthip, Arpaporn Yodmeeklin, Niwat Maneekarn

**Affiliations:** 1 Department of Microbiology, Faculty of Medicine, Chiang Mai University, Chiang Mai, 50200 Thailand; 2 Center of Excellence in Emerging and Re‑Emerging Diarrheal Viruses, Chiang Mai University, Chiang Mai, 50200 Thailand; 3 Youjiang Medical University for Nationalities, Baise, 533000 Guangxi China

Correction to: *Scientific Reports* https://doi.org/10.1038/s41598-021-02745-7, published online 01 December 2021

The original version of this Article contained errors in Table 1, where the percentage total (%) of “No. of HAstV co-infection” was incorrect.

Incorrect:

**Table:** 

Years	No. of specimen tested	No. of HAstV positive (%)	No. of HAstV single infection (%)	No. of HAstV co-infection (%)	No. and patterns of HAstV co-infection				
					RV	NoV	SaV	EV	≥ 2 viruses^a^
2017	257	8 (3.1)	3 (37.5)	5 (45.5)	-	1	1	1	2

Correct:

**Table:** 

Years	No. of specimen tested	No. of HAstV positive (%)	No. of HAstV single infection (%)	No. of HAstV co-infection (%)	No. and patterns of HAstV co-infection				
					RV	NoV	SaV	EV	≥ 2 viruses^a^
2017	257	8 (3.1)	3 (37.5)	5 (62.5)	-	1	1	1	2

The original Article has been corrected.

